# Clinical scale labeling of T cell immunotherapy for MRI cell tracking

**DOI:** 10.1186/2051-1426-3-S2-P389

**Published:** 2015-11-04

**Authors:** Brooke M Helfer, William Shingleton, Shannon Eaker, Charles O'Hanlon, Eric Ahrens

**Affiliations:** 1 Celsense, Inc, Pittsburgh, PA, USA; 2 GE Healthcare Life Sciences, Amersham, UK; 3 GE Healthcare Life Sciences, Knoxville, TN, USA; 4 University of California at San Diego, La Jolla, CA, USA

## 

Leukocyte immunotherapies have made a great progress and hold much promise in the treatment of cancer. Specifically, in the case of B cell malignancies (such as Acute Lymphoblastic Leukemia, or ALL), CAR (chimeric antigen receptor) and TCR (T cell receptor) cell therapies have demonstrated encouraging clinical results. As we begin to look into targeting solid tumors with TCR and CAR T cells, the hurdle of being able to select a suitable target and achieving successful cellular delivery/homing to the site of disease remains. Additional challenges include the preparation and administration of a therapeutic dose involving minimums such as 1X10^9^ cells per patient. Here we demonstrate the application of a clinically applicable perfluorocarbon (PFC) tracer agent that enables the migration and persistence of cellular therapies to be noninvasively imaged by 19F MRI. Using a general T cell expansion protocol, we show that adding a cellular label does not alter the viability, growth curves, or release characteristics of T cell therapies, and most importantly, that the labeling process is able to be performed at a large clinical scale without detriment to the product. By pairing the PFC signal with conventional proton MRI from the same imaging session, the images are able to be overlaid, allowing cells to be traced to their anatomical location. With nominal exogenous fluorine naturally present in tissue, labeled cells appear with little background. Further animal biodistribution studies and clinical patient scans of labeled cells demonstrate both the migratory capacity of cells between 2 and 24 hours post-administration and the sensitivity of this method at clinically relevant scan times. The MRI tracking capabilities, safety profiles, scalability, and clinical sensitivity of this method demonstrate the ability of 19F to be used in additional clinical applications in order to visualize the spatial fate of cellular therapeutics.

